# Effect of emerging digital technologies and methodologies combined with incentives on HbA1c in patients with type 2 diabetes mellitus: study protocol for a parallel, open randomized controlled trial

**DOI:** 10.1186/s13063-024-07950-z

**Published:** 2024-02-01

**Authors:** Shao Zhong, Jingyi Jiang, Hongying Liu, Ying Pan

**Affiliations:** 1 Department of Endocrinology, First People’s Hospital of Kunshan Affiliated With Jiangsu University, Kunshan, 215300 China; 2 Hangzhou Kang Ming Information Technology Co., Ltd, Hangzhou, 310000 China

**Keywords:** Behavioral economics, Digital health, Type 2 diabetes mellitus

## Abstract

**Background:**

Type 2 diabetes mellitus (T2DM) is a common metabolic disease that requires long-term management and treatment. Digital intervention, as an emerging medical model, has been widely used in the treatment of T2DM patients. Behavioral economics theory provides a favorable perspective for studying the effect of digital intervention because it can reveal the decision-making mechanisms behind human behavior and provide more effective interventions for digital intervention. The purpose of this trial is to evaluate the impact of behaviorally based digital intervention on T2DM patients’ HbA1c, self-monitoring of blood glucose (SMBG) testing rate, diabetes self-efficacy, and other indicators compared to conventional treatment.

**Methods:**

This trial is a prospective randomized controlled trial conducted at the First People’s Hospital of Kunshan City from April 1, 2023, to December 31, 2024, with a follow-up period of 3 months. The specific randomization method was established and implemented through the EDC clinical trial center’s randomization system. We will measure and collect baseline data from three groups, including Group A: digital intervention + virtual incentives + conventional treatment, Group B: digital intervention + physical incentives + conventional treatment, and Group C: conventional treatment. HbA1c, weight, SMBG testing rate, diabetes self-efficacy, and diabetes-related medical expenses will be recorded at baseline, 1 month, 2 months, and 3 months for all three groups. The Shapiro-Wilk test will be used to test for normality, and Pearson correlation analysis will be used for correlation analysis. Dropouts will be analyzed separately. Analysis of variance or exact probability calculation will be used to compare demographic data and other baseline indicators.

**Discussion:**

This study is a novel clinical trial that integrates multiple disciplines (economics and medicine) and uses digital technology to deliver the intervention. Most published studies were offline interventions based on behavioral economics theory, but very few were on online interventions for T2DM patients. This study has both novelty and social value.

**Trial registration:**

Chinese Clinical Trial Registry ChiCTR2300070753. Registered on 2023/04/22.

**Supplementary Information:**

The online version contains supplementary material available at 10.1186/s13063-024-07950-z.

## Introduction

Diabetes is a common chronic disease with high-risk complications, resulting in a heavy medical burden. According to a recent report by the International Diabetes Federation (IDF), the number of adults aged 20–79 with diabetes in China is 140.9 million, accounting for about a quarter of the total number of people with diabetes worldwide; by 2045, this number will grow to 174.4 million, ranking first in the world [1]. Type 2 diabetes mellitus (T2DM) is the most common form of diabetes, accounting for approximately 90% of all cases. In 2019, direct health care expenditures due to T2DM have reached $760 billion [2]. The prevalence of T2DM has remained high for many years. Unhealthy diet and low physical activity appear to be the causal factors behind it [3]. Several studies have highlighted the role of lifestyle interventions in reducing the incidence and progression of T2DM [4, 5]. Effective lifestyle management in people with diabetes can help to delay progression, reduce the risk of complications, and improve quality of life. However, a lack of timely communication between doctors and patients reduces the continuity of intervention and monitoring, resulting in poor management of diabetic patients. Therefore, we are attempting to explore a new approach and method to provide efficient, continuous, and personalized lifestyle interventions for diabetic patients.

Digital healthcare is a new form and model that uses digital technology and information and communication technology (ICT) to improve healthcare [6], covering the entire healthcare process from prevention and early diagnosis to treatment and rehabilitation, including aspects of medical research, clinical practice, and patient management. Electronic medical record systems, medical imaging and diagnosis, mobile medical applications, and health management and monitoring are the main forms of digital health applications in healthcare. Some studies have shown that the adoption of mobile health apps has been a good driver in helping people with diabetes with diet, exercise, and lifestyle management [7, 8]. Another meta-analysis showed that mobile app-assisted self-care interventions had a significant effect on HbA1c levels, fasting blood glucose levels, and waist circumference control in patients with T2DM [9]. Overall, previous studies have shown that mHealth technology and platform design have great potential to achieve better healthcare outcomes.

The long-term treatment process of chronic diseases relies heavily on patient self-management. An observational study found that among various mobile applications, patients with T2DM exhibited the highest level of engagement only in monitoring daily physical activity using accelerometers [10]. This may be attributed to the passive nature of data collection and transmission through accelerometer-type devices. In this study, we propose for the first time the idea of designing medical applications using behavioral economics and cognitive behavior therapy (CBT) as methodological approaches to design medical applications in order to improve the attractiveness of everyday tasks and increase organizational effectiveness. Behavioral economics theory suggests that human decision-making and behavior are influenced by emotions and social factors, allowing designers to leverage these factors to influence human decision-making. Adding gamification design based on this theory can significantly improve patient engagement and compliance in digital therapy [11, 12]. Previous studies have shown that a small financial incentive coupled with a written reminder can increase patient participation at HbA1c screening [13]. The incentive setting of a reminder letter and a $6 gas card increased the average number of HbA1c tests for T2DM patients from 2.7 to 3.3 over 2 years [14]. CBT, on the other hand, helps mobile applications establish the correct system of cognition and behavior. Using this framework, we aim to investigate the effectiveness of incentive design mechanisms in gamification for managing patients with T2DM by comparing the effectiveness of monetary rewards and non-monetary rewards. This research will contribute to expanding the existing theories of gamified information systems.

Through this study, we aim to collect data on the health activities of patients with T2DM, such as step count and dietary habits, as well as changes in HbA1c levels, in order to elucidate the impact of a novel digital intervention approach on T2DM patients. Additionally, we will further explore the influence of incentive mechanisms on patients’ health behaviors.

### Evidence before this study

Diabetes is a chronic disease that seriously threatens the health of patients. However, the current management mode has a poor control rate for diabetes. Digital intervention is a new means of daily management, monitoring, and intervention of patients using digital monitoring technology and information communication technology. Gamification and social incentive measures are the main reasons why digital intervention platforms attract patients to use them [15]. We searched PubMed for studies using behavioral economics-based digital intervention in T2DM patients from November 2016 to December 2022. The search terms included T2DM, digital intervention, behavioral economics, and gamification. Medical subject headings (MeSH) were used to include synonyms. According to our search, some studies have shown that digital intervention is effective in strengthening diabetes self-management education and blood glucose control in adult T2DM patients [16]. However, almost no studies based on interdisciplinary theoretical integration have been published in the T2DM population. We identified two randomized controlled trials evaluating the effectiveness of digital intervention platforms in T2DM patients. The results showed that compared with the control group (*n*=120), the blood glucose control of patients in the digital monitoring group (*n*=120) was significantly improved during the follow-up period until the end of the study [17]. There was no difference in the number of adverse events and health-related quality of life between the two groups. Another multicenter randomized controlled trial found that for every additional day of mobile app use, the HbA1c level of participants (*n*=57) decreased by 0.016 points (95% CI: −0.03 to −0.003). However, the use of the application varied among the trial sites [18].

### Added-value of this study

Previous studies mainly focused on applying digital intervention platforms in hospitals to improve the blood glucose management of inpatients with diabetes by nurses and doctors. However, information on the impact of digital intervention on T2DM patients outside the hospital is limited. Our study aims to evaluate the effectiveness of behavior economics-based digital intervention on HbA1c, SMBG testing rate, and diabetes self-efficacy in T2DM patients. During the study, we added a reward mechanism. By comparing the effects of tangible and virtual incentives on patients’ SMBG testing rate and self-efficacy, we studied the effect of incentive design mechanism in gamification on managing T2DM patients.

## Materials and methods

### Objectives

Aim1: To assess the effect of behaviorally informed digital intervention on HbA1c levels, prevalence of SMBG detection, weight, diabetes self-efficacy, and diabetes-related expenses in patients with type 2 diabetes compared to standard treatment.

Hypothesis 1a: Evaluate the changes in HbA1c levels in the intervention and control groups. At 3 months, there will be significant differences in HbA1c levels prevalence of SMBG detection, weight, diabetes self-efficacy, and diabetes-related expenses among the three groups: A (digital intervention + virtual incentives + standard treatment), B (digital intervention + tangible incentives + standard treatment), and C (standard treatment).

### Study design and setting

This study is a single-center, randomized, controlled, open-label, parallel-group superiority trial. Patients were randomly assigned in a 1:1:1 ratio to evaluate the efficacy of behavioral economics-based digital intervention on HbA1c, SMBG testing rate, and diabetes self-efficacy in patients with T2DM. The study protocol followed the Standard Protocol Items: Recommendations for Interventional Trials (SPIRIT) guidelines [19]. This study will be conducted at the First People’s Hospital of Kunshan City, Jiangsu Province, China, from April 1, 2023 to December 31, 2024.

### Inclusion criteria

Patient inclusion criteria are as follows: (1) aged 18–65 years old; (2) diagnosed with T2DM according to the 1999 WHO criteria, and with a disease duration of > 6 months; (3) stable treatment for diabetes for ≥2 months (e.g., metformin ≥ 1000mg/day or maximum tolerated dose), with 7.0% ≤ HbA1c ≤ 10.5%; (4) able to use a smartphone without barriers and have basic Chinese literacy and simple arithmetic skills (education requirement: primary school graduation level and above); (5) voluntarily sign an informed consent form and agree to strictly follow the requirements of this protocol.

### Exclusion criteria

The exclusion criteria for patients are as follows: (1) use of traditional Chinese medicine or herbal medicine with hypoglycemic effects within 2 months prior to screening; (2) currently receiving chronic systemic corticosteroid therapy (> 2 weeks), or use of corticosteroids within 4 weeks prior to screening (except for topical, intraocular, intranasal, or inhaled administration); (3) occurrence of 2 or more episodes of ketoacidosis, hyperosmolar state, or lactic acidosis within 6 months prior to screening; (4) history of severe hypoglycemic events within 6 months prior to screening, defined as the presence of neurological symptoms of hypoglycemia that require assistance from others to recover, or previous lack of knowledge about hypoglycemia or inadequate understanding of hypoglycemic symptoms, and the researcher deems that the subject is unable to communicate and understand hypoglycemic symptoms and appropriate treatment should also be excluded from the study; (5) severe chronic complications of T2DM, such as diabetic foot; (6) history of acute or chronic pancreatitis; (7) previous or current proliferative diabetic retinopathy or diabetic macular edema requiring urgent treatment; (8) previously diagnosed autonomic neuropathy manifested as urinary retention, resting tachycardia, orthostatic hypotension, or diabetic diarrhea; (9) occurrence of acute myocardial infarction, unstable angina, coronary artery bypass grafting, percutaneous coronary intervention (excluding diagnostic angiography), transient ischemic attack, cerebrovascular accident, decompensated heart failure classified as NYHA class III or IV within 6 months prior to screening; (10) concomitant acute non-cardiac disease, such as infection, renal failure, and hyperthyroidism; (11) concomitant other severe cardiovascular diseases, including moderate to severe stenotic valvular heart disease, hypertrophic cardiomyopathy or other forms of outflow tract obstruction, acute myocarditis, pericarditis, active endocarditis, suspected or known ruptured aneurysm, and acute pulmonary embolism or pulmonary infarction; (12) concomitant neurological, muscular, skeletal, or rheumatic diseases that may worsen with exercise; (13) mental illness that cannot live independently or cooperate with treatment; (14) lactation, pregnancy, or planned pregnancy; (15) patients deemed unsuitable for inclusion in the study by the researcher.

### Interventions

To compare the effects of digital intervention based on behavioral economics theory on HbA1c, SMBG testing rate, and diabetes self-efficacy in patients with T2DM, conventional treatment was used as a control intervention. Conventional treatment was administered according to the T2DM conventional drug treatment method recommended by clinical guidelines. Conventional drugs include but not limited to metformin, sulfonylureas, glinides, α-glycosidase inhibitors, TZDS, DPP-4i, SGLT2i, GLP-1RA, and insulin. Digital intervention refers to the use of WeChat mini-program in smartphones to provide patients with out-of-hospital coordinated management based on cognitive behavioral therapy, guiding patients to improve their health behaviors in coping with diabetes and enhance the level of blood glucose self-management. The main content includes disease knowledge courses, auxiliary training courses (diabetic diet essentials), psychological counseling courses, course Q&A games, and relevant behavior records (daily attendance, medication check-ins, daily sugar control diet punch card). From day 1 to 1 month of the study is the first cycle, to 2 months is the second cycle and to 3 months is the third cycle.

After completing a single cycle of digital intervention, the subjects who receive digital intervention can receive virtual and tangible incentives respectively. On the first day of each cycle, the incentive content to be received at the end of the cycle will be reminded during follow-up. The subjects need to provide self-recorded and completed SMBG diaries to the researchers to receive incentives after check-in. The virtual incentive is a healthy behavior learning badge that lights up each cycle, including bronze, silver, and gold, which are awarded in turn according to the user’s completion (≥70%, ≥80%, ≥90%) judged by the researcher. The tangible incentive is a health behavior auxiliary tool provided each cycle, including an electronic medication box, a weight scale, and a smart bracelet, which are awarded in turn according to the user’s completion (≥70%, ≥80%, ≥90%) judged by the researcher.

### Criteria for discontinuing or modifying allocated interventions

Participants will not be considered as having withdrawn from the study in the following cases, and they will be eligible for protocol evaluation unless they withdraw their consent for assessments:

1. If the participant requests to discontinue the program treatment;
2. If the participant is unable to continue the program treatment due to adverse events;
3. When the researcher considers that the risks of continuing the program treatment outweigh the benefits for any reason; and
4. For any of the other reasons, the researcher deems it unsuitable to continue the program treatment.

### Adherence

The intervention group participants will use WeChat mini-program to log in and complete the intervention sessions according to the plan. The completion of each course will be tracked through the mini-program. The “diary check-in” function in the mini-program will provide positive and constructive feedback to participants to improve compliance. Investigators will be able to “check-in” from the dashboard, and regularly track their learning, medication, and lifestyle blood sugar control behaviors.

### Ancillary for post-trial care

As for the nature of digital patient education, participants are unlikely to be harmed. Once a risk of harm is identified, the principal investigator will intervene to reduce the risk of harm. If any harm is discovered, the research team will appropriately compensate participants as necessary.

### Outcomes

#### Primary outcome measures

The primary endpoint is to compare the difference in HbA1c levels among the three groups at the 3-month follow-up. After enrollment, the HbA1c levels will be recorded at baseline and at the end of the first, second, and third digital intervention cycles.

#### Secondary outcome measures

1. At the 3-month follow-up, the difference in SMBG testing rate among the three groups will be compared;
2. At the 3-month follow-up, the difference in weight among the three groups will be compared;
3. At the 3-month follow-up, the difference in diabetes self-efficacy among the three groups will be compared;
4. At the 3-month follow-up, the difference in diabetes-related diagnosis and treatment expenses among the three groups will be compared.

### Participant timeline

The schedule of the participants is shown in the Table 1.

**Table 1:** The schedule of the participants

Period	Baseline screening	Follow-up period			
Interview	1	2	3	4	5
Days	-7~-1	1	28 ± 5	56 ± 5	84 ± 5
Informed consent	√				
Baseline characteristics	√				
Physical exams/urinalysis/ Kidney and liver functions	√				
H1Abc	√				√
Inclusion exclusion criteria review	√				
Random allocation		√			
Digital intervention^1^		√	√	√	√
Conventional therapy		√	√	√	√
Incentives^2^			√	√	√
SMBG testing rate^3^			√	√	√
Weight^4^			√	√	√
Scale evaluation^5^		√	√	√	√
Medical expenses^6^		√	√	√	√
Adverse events evaluation		√	√	√	√

(1) Digital intervention: implementation of the WeChat mini-applet. (2) Incentives: patients will be given incentive content at the end of each monthly follow-up. (3) Calculation of testing rate: self-blood glucose monitoring once every morning on an empty stomach, continuous monitoring for 3 months, detection rate = actual measurement times/expected measurement times, and data recorded in the SMBG diary card. (4) Weight: measured at the clinic during each follow-up visit. (5) Scale evaluation: completed the scale evaluation under the doctor’s instructions. (6) Diagnosis and treatment expenses: during the 3-month period, all medical expenses related to diabetes diagnosis and treatment, including outpatient and inpatient visits, will be recorded

### Sample size

This study employs three groups with a 1:1:1 ratio. The primary research indicator is HbA1c, where *α*=0.05, *β*=0.2, and considering a 20% dropout rate, the minimum sample size for a single group is 100 cases. Therefore, a total of at least 300 cases should be included in the three groups.

### Recruitment

We will recruit patients at the First People’s Hospital of Kunshan in Jiangsu Province, China. Once recruited, the study staff will guide patients through the process of learning digital intervention operations/diary card recording, and confirm the content and frequency of digital intervention and the requirements for diary card recording with the patients.

### Sequence generation

In this randomized controlled trial, a simple randomization method was used to allocate participants to three groups with a 1:1:1 allocation ratio. Group A: digital intervention + virtual incentives + conventional treatment, Group B: digital intervention + physical incentives + conventional treatment, and Group C: conventional treatment. A central randomization system for the EDC clinical trial is established and implemented. Randomizers at the trial center will access the system with an account number and password, enter basic information about the subject patients and generate a randomization number for follow-up.

### Concealment mechanism

Allocation concealment will be ensured by the following mechanism. Staff will log into the EDC Trial Data Cloud and enter information for participants. The EDC trial data system will randomly assign participants to any group in a 1:1:1 ratio. Once the necessary baseline information has been entered, a patient summary will be generated. To evaluate the data, an account and password will be required.

### Implementation

The allocation sequence is generated by the EDC centralized randomization system. The research assistant will be responsible for patient registration. The Trial Management Committee (TMC) will assign interventions to participants.

### Assignment of interventions

Participants and investigators will be informed about the interventions assigned to them in this open-label trial. Due to the nature of the patient-reported outcome indicators, not all outcome evaluations will be blinded. Assignment will not be known to statisticians during the analysis. As this is an open-label trial, no blind-breaking procedures will apply.

### Data collection

Data will be collected at the study site using EDC. Data will be collected at a total of 5 time points: baseline, intervention period day 1, day 28±5, day 56±5 and day 84±5. The data collected will be stored in the form of an electronic CRF. Investigators will be informed of any changes made by the CRC and the system will automatically perform a system/edit check. Authorized investigators will be given access to the study computer and will evaluate the data throughout the course of the study. If any missing data is detected through the EDC system, the investigator will contact the participant by telephone.

### Data management

Internal monitoring includes monitoring of appropriate informed consent documentation/records, eligibility criteria, data quality, etc. Monitoring is usually carried out by relevant parties involved in the study to identify problems and improve the process. When data is analyzed, a unique identifier is generated to ensure the security of the data. Information on incorrect or missing data is sent to the Data Manager (DM) in the Data Query Report. The DM who receives the review will check the original records to identify corrections. The original documents and signed informed consent forms will be stored in a locked filing cabinet.

### Confidentiality

Personal information will be collected, shared and maintained in accordance with the International Conference on Harmonisation Good Clinical Practice Guidelines (ICH GCP).

### Statistical methods

All data will be entered separately by 2 members and analyzed after confirming complete agreement of data. SPSS 22.0 will be used for statistical analysis. All measures will be tested for normality using the Shapiro-Wilk method, and data that conform to a normal distribution will be statistically described using the mean ± standard deviation; data that do not conform to a normal distribution will be statistically described using the median (P25, P75), and correlation will be analyzed by Pearson correlation analysis, with differences considered statistically significant at P<0.05. Statistical descriptions of the different groups will be made using the mean, standard deviation, median, minimum, and maximum values for the different groups of measures. Statistical description of the count data of the different groups will be made using frequency (composition ratio). Changes before and after the intervention in each group will be calculated using exact probability or non-parametric tests. The analysis of dropout will statistically describe the actual number of subjects enrolled, the number of dropouts, and the number of dropouts one by one and analyze the specific reasons for dropout and dropout. Analysis of the balance of underlying values will use ANOVA or exact probability calculations to compare demographic information with other indicators of underlying values to measure the extent to which each group is in balance.

All patients who have received the behavioral economics-based digital intervention for at least 4 weeks will be included in the full analysis set (FAS). Depending on the timing of the survey, the analysis set following the study protocol (per-protocol, PP) will consist of patients who have completed a 3-month follow-up examination. The FAS will be used as the basis for the preliminary analysis. If the difference between the FAS and PP samples is greater than 10%, the analysis will be repeated for the PP analysis set. The safety population includes patients with FAS. Unless otherwise stated, all analyses will be performed on both the FAS and PP analysis sets. No additional analyses will be performed.

Missing values will not be replaced or added. All analyses will be based solely on observed cases. Patients lost to follow-up will be considered to have dropped out of treatment. Only PP patients will be used in sensitivity analyses to test the impact of this assumption. No genetic or molecular analysis will be performed in this trial or future use.

### Composition of the coordinating center and trial steering committee

Design and conduct of the study: preparation of the program and amendments; preparation of the Investigators’ Brochure (IB) and CRF; study to be published; composition of the TMC membership.

Steering Committee (SC): final protocol; recruit patients and communicate with the principal investigator; review the progress of the trial and agree adjustments to the protocol and/or investigator manual if necessary to help the study run smoothly. The steering group will include all principal investigators.

Trial Management Committee (TMC): (Principal Investigator, Study Physician, Administrator): study planning; conducts steering committee meetings; reports adverse events to the Chinese National Adverse Reaction Monitoring Centre; responsible for trial master file preparation; budget management and contract difficulties with individual centers; randomization; data validation; TMC will review 3 monthly feedback forms and arrange site visits; data manager: data entry and maintenance of clinical trial IT systems; data validation.

Lead Investigators: recruitment; data collection; CRF completion, study patient follow-up, and adherence to study protocol and IB.

### Composition of the data monitoring committee, its role, and reporting structure

There will be no data monitoring committee for this study because the intervention in this study is considered a low-risk intervention.

### Interim analyses

There will be no interim analysis.

### Adverse event reporting and harms

Any adverse events that occurred between the time the subject signed the informed consent and was enrolled in the study and the end of the study, regardless of whether there was a causal relationship with the intervention. Any medical condition or clinically significant laboratory abnormality occurring prior to the administration of the intervention on day one is considered pre-existing and must be documented on the case report form. All AEs occurring after the administration of the intervention up to the last day of the study (including follow-up, the pause period of the study) must be recorded as AE subsets in the appropriate CRF section. When completing the adverse event form in the CRF, the investigator will use a scale of “1 to 5” to describe the severity of the adverse event. To standardize the criteria, the intensity of the adverse event will be judged by reference to the CTCAE v3.0 grading definitions: Grade 1 (mild, asymptomatic, or with mild signs; clinical or diagnostic observations only; no intervention required), Grade 2 (moderate, requiring minimal, local or non-invasive treatment, age-appropriate instrumental limitation of activities of daily living), Grade 3 (severe or clinically significant but not immediately life-threatening; hospitalization or prolonged hospitalization; disabling; limited ability to perform activities of daily living), Grade 4 (life-threatening, requiring urgent treatment), Grade 5 (death). The type and frequency of any adverse event will be reported to the Chinese National Adverse Reaction Monitoring Centre.

### Frequency and plans for auditing trial conduct

The audit process will comply with ICH GCP and regulatory requirements. Twice yearly audits will be conducted by the Hospital Ethics Committee and the audit process will be independent of the Sponsor. The SC will decide on any revisions to the program. The revised protocol will also be submitted to the Medical Ethics Committee of Kunshan First People’s Hospital and reported to the participants as and when required. The results of this study will be presented to the public at an academic conference. Authorship will be determined by the Steering Group. The order of authorship will be determined by the contribution of each member.

## Discussion

This paper describes in detail a parallel randomized controlled trial designed to assess the impact of digital intervention based on behavioral economics theory on indicators such as HbA1c, SMBG detection rates, and diabetes self-efficacy in patients with T2DM. Digital health care has been widely used in health systems, particularly during the COVID-19 pandemic, and to reduce the risk of virus transmission between patients and clinicians, many health systems have rapidly converted more than 70% of outpatient cases to digital care via telephone or video transmission [20, 21]. However, concerns remain that chronic disease management delivered through digital processes may be of poor quality, difficult for patients to use proactively and spontaneously, and may exacerbate health disparities. To improve digital chronic disease management even more, we think that digital intervention based on behavioral economics theory is a feasible way to improve HbA1c levels in patients with T2DM. Such digital interventions integrate multidisciplinary theories. We hypothesized that digital intervention based on behavioral economics theory would be effective in improving SMBG detection rates and diabetes self-efficacy in T2DM patients. The significance of this study is that, until now, research on digital intervention and patients with T2DM has been limited to the hospital setting. No studies have yet reported the effectiveness of digital intervention based on behavioral economics theory in T2DM patients outside the hospital. As behavioral economics theory highlights that people are influenced by cognitive biases and emotional factors when making decisions, the intervention process will be gamified and is expected to increase patients’ interest in using it. In addition, this study may demonstrate that a digital intervention based on behavioral economics theory can reduce the financial burden on T2DM patients. Moreover, there are some limitations to this study. This study excluded T2DM patients treated with multi-drug combinations and only included T2DM patients who were stably treated with mono-therapy. Future studies may involve a larger group of patients. Secondly, due to the nature of the study, it was not possible to use blinding among participants and staff (excluding statisticians) during digital administration, which may have biased the expected effect of treatment.

### Trial status

The present protocol is version 1.0, dated September 26, 2022. Participant recruitment has began on April 15, 2023, and is expected to be completed by December 15, 2023.

### Supplementary Information

**Additional file 1.** SPIRIT Checklist for Trials

## Data Availability

Not applicable.

## Abbreviations

T2DM: Type 2 diabetes mellitus
HbA1c: Glycosylated hemoglobin
SMBG: Self-monitoring of blood glucose
EDC: Electronic data capture
IDF: International Diabetes Federation
ICT: Information and communication technology
CBT: Cognitive behavior therapy
MeSH: Medical subject headings
SPIRIT: Standard Protocol Items: Recommendations for Interventional Trials
WHO: World Health Organization
TMC: Trial Management Committee
CRF: Case Report Form
CRC: Clinical research coordinator
DM: Data Manager
ICH GCP: International Conference on Harmonisation Good Clinical Practice Guidelines
FAS: Full analysis set
PP: Per-protocol
IB: Investigators’ Brochure
SC: Steering Committee
TMC: Trial Management Committee
AE: Adverse event
CTCAE: Common Terminology Criteria for Adverse Events
COVID-19: Coronavirus disease 2019
